# MicroRNA-133a inhibits proliferation and invasion, and induces apoptosis in gastric carcinoma cells via targeting fascin actin-bundling protein 1

**DOI:** 10.3892/mmr.2021.12185

**Published:** 2021-06-01

**Authors:** Chen Lai, Zihua Chen, Ruixin Li

Mol Med Rep 34: 1473-1478, 2015; DOI: 10.3892/mmr.2015.3545

Following the publication of this paper, it was drawn to the Editors’ attention by a concerned reader that certain of the flow cytometric data in the article (featured in Fig. 4B) were strikingly similar to data that had appeared in different form in another article by different authors.

Owing to the fact that the contentious data in the above article had already appeared in different form in another article prior to its submission to Molecular Medicine Reports, the Editor has decided that this paper should be retracted from the Journal. After having been in contact with the authors, they agreed with the decision to retract the paper. The Editor apologizes to the readership for any inconvenience caused.

